# Histone Deacetylases: Transcriptional Regulation and other Cellular Functions

**DOI:** 10.1038/sj.bjc.6603698

**Published:** 2007-04-03

**Authors:** G Garcia-Manero

**Affiliations:** 1 University of Texas MD Anderson Cancer Center, Houston, Texas, USA

As a leukemia physician with an interest in epigenetic therapy of cancer, I found ‘Histone deacetylases’ not only timely but very informative. The term epigenetic alterations refers to biochemical modifications of the chromatin, at the DNA, protein and RNA levels, that have an impact, among several other functions, on gene transcription regulation. Interest in two of these modifications, DNA methylation and alterations of histone code, stands not only from their fundamental molecular implications, but also from a therapeutic perspective. These two modifications are the target of anticancer drugs with either hypomethylating or histone deacetylase (HDAC) inhibitory activity. Indeed, three drugs, 5-azacitidine, 5-aza-2^′^-deoxycitine and suberoylanilide hydroxamic acid (SAHA), have been approved for clinical use in the United States. The first two drugs are hypomethylating agents with activity in myelodysplastic syndromes and other leukaemias. The third drug, a potent HDAC inhibitor, was approved for cutaneous lymphoma, and it also has clinical activity in other disorders.

HDACs are a very complex group of proteins. They are divided into three families: Class 1 (HDAC 1, -2, -3 and -8); Class 2 (HDAC 4, -5, -6, -7, -9, -10 and -11); and Class 3 (SIRT 1–7). The function of these groups of proteins is now starting to be understood. It is also clearly emerging that HDACs can regulate acetylation of other nonhistone proteins, adding significant complexity to this field. Furthermore, to date, the anticancer mechanisms of action of HDAC inhibitors are unknown. It is also not known whether induction of histone acetylation, that is well documented to occur *in vivo* after administration of HDAC inhibitors, is a key mediator of their anticancer activity. Therefore, the importance and need of a comprehensive book source for investigators and individuals with interest in this area.

This text is divided into four main chapters focusing on each HDAC class family and a final chapter on HDAC inhibitors. Subsequently, each chapter is divided into subchapters addressing specific HDACs or topics of interest. Most of these subchapters are of interest, and although there is some degree of redundancy, common in this type of multiauthor books and mainly in the introductory remarks, a wealth of data, both historic and current, is presented. Most chapters are succinct and to the point, and this makes for easy reading. More importantly, all chapters come with a very extensive and relevant set of references that, in many cases, are as useful as the text itself. In particular, chapter three, which relates to class 3 HDACs, is very useful as this is a relatively newer area of knowledge and broad potential clinical interest. The figures and tables are small in number and of limited value, specifically those tables/figures of protein/gene sequence alignments.

Unfortunately, the weakest area of the book review is the one that relates to the translational applications, in particular against cancer, of the information provided here. For instance, there is little in terms of potential Sirtuin inhibitors. Furthermore, only two subchapters focus on HDAC inhibitors. These chapters are not sufficiently updated (probably because of the delays intrinsic to this type of publication). The book would have significantly benefited from a more systematic review of the clinical activity and studies with this class of drugs.

Despite this limitation, I strongly recommend this text, especially for those (like myself) who want and need a condensed, well-referenced guide to the area of HDACs. In that sense, this book more than fulfils its objective.

